# Advanced binder-free electrodes based on CoMn_2_O_4_@Co_3_O_4_ core/shell nanostructures for high-performance supercapacitors†

**DOI:** 10.1039/c8ra06289g

**Published:** 2018-09-10

**Authors:** Xiaobo Chen, Xiao Liu, Yongxu Liu, Yameng Zhu, Guoce Zhuang, Wei Zheng, Zhenyu Cai, Peizhi Yang

**Affiliations:** School of New Energy and Electronic Engineering, Yancheng Teachers University Yancheng 224051 PR China chenxbok@126.com; Key Laboratory of Education Ministry for Advance Technique and Preparation of Renewable Energy Materials, Yunnan Normal University Kunming 650500 PR China pzhyang@hotmail.com

## Abstract

Three-dimensional (3D) hierarchical CoMn_2_O_4_@Co_3_O_4_ core/shell nanoneedle/nanosheet arrays for high-performance supercapacitors were designed and synthesized on Ni foam by a two-step hydrothermal route. The hybrid nanostructure exhibits much more excellent capacitive behavior compared with either the pristine CoMn_2_O_4_ nanoneedle arrays alone or Co_3_O_4_ nanosheets alone. The formation of an interconnected pore hybrid system is quite beneficial for the facile electrolyte penetration and fast electron transport. The CoMn_2_O_4_@Co_3_O_4_ electrode can achieve a high specific capacitance of 1627 F g^−1^ at 1 A g^−1^ and 1376 F g^−1^ at 10 A g^−1^. In addition, an asymmetric supercapacitor (ASC) was assembled by using the CoMn_2_O_4_@Co_3_O_4_ core/shell hybrid nanostructure arrays on Ni foam as a positive electrode and activated carbon as a negative electrode in an aqueous 3 M KOH electrolyte. A specific capacitance of 125.8 F g^−1^ at 1 A g^−1^ (89.2% retention after 5000 charge/discharge cycles at a current density of 2 A g^−1^) and a high energy density of 44.8 W h kg^−1^ was obtained. The results indicate that the obtained unique integrated CoMn_2_O_4_@Co_3_O_4_ nanoarchitecture may show great promise as ASC electrodes for potential applications in energy storage.

## Abstract 2

CoMn_2_O_4_@Co_3_O_4_ core/shell arrays on Ni foam exhibit outstanding electrochemical performance for asymmetric supercapacitors with respect to high specific capacitance and high cycling stability.

## Graphical abstract

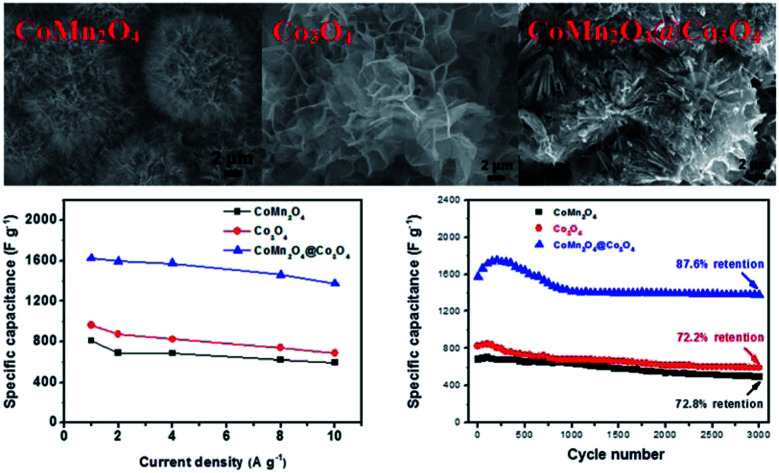

## 1. Introduction

Supercapacitors have attracted a lot of attention due to their ultrafast charge–discharge capability, reversibility, safe operation, long cycle life, high power density and environmental friendliness.^1–3^ Supercapacitors are usually divided into two categories according to their energy storage behavior: electrical double-layer capacitors (EDLCs) and pseudocapacitors.^4,5^ Among them, pseudocapacitors exhibit higher specific capacitance due to the fast and fully reversible faradaic redox reactions at the interface between the electrode and electrolyte. As electrode materials for pseudocapacitors, transition metal oxides including Co_3_O_4_ and MnO_2_ and their related compounds have been widely studied.^6–12^

Recently, manganese-based transitional metal oxides (such as CoMn_2_O_4_) have emerged as a promising electrode material for supercapacitors due to their high reversible capacities and eco-benignity.^13^ Jiang *et al.* synthesized hierarchical nanosheets of CoMn_2_O_4_ on Ni foam using a hydrothermal method, which exhibited a high capacitance of 840 F g^−1^ at 10 A g^−1^ and retained 102% of its initial capacitance after 7000 cycles.^14^ Ren *et al.* fabricated flowerlike microspheres of CoMn_2_O_4_, and it showed the specific capacitance of 188 F g^−1^ at 1 A g^−1^ with a capacitance retention of 93% after 1000 cycles.^15^ However, the experimental values of the capacitance are appreciably lower than the theoretical ones as has already been observed. There is a great need and a challenge to enhance the capacitive performance of CoMn_2_O_4_. Three-dimensional (3D) nanostructured electrodes have been intensively studied as attractive candidates for electrodes of high-performance supercapacitors due to their unique morphological architectures and super electrochemical properties,^16–18^ and especially unique porous 3D core/shell nanostructures usually exhibit novel physicochemical properties.^19–21^ Therefore, intensive research efforts have been expended to design 3D core/shell nanostructured electrodes to shorten diffusion length of electrolytes to interior surfaces. Cai *et al.* fabricated 3D Co_3_O_4_@NiMoO_4_ core/shell nanowire arrays *via* a facile two-step hydrothermal method. The material showed excellent electrochemical performance with a remarkable areal capacitance of 5.69 F cm^−2^ (1094 F g^−1^) at a current density of 30 mA cm^−2^.^22^ Liu *et al.* fabricated Co_3_O_4_@MnO_2_ core/shell hierarchical nanowire arrays and showed remarkably improved electrochemical performance (about 4 to 10 times increase in areal capacitance with respect to single Co_3_O_4_ array).^23^ Therefore, taking into account the above-mentioned consideration, it is therefore of great possibility to the rational design and fabrication of CoMn_2_O_4_@Co_3_O_4_ core/shell hybrid nanostructure arrays as electrode materials with combined properties of large areal capacitance and rate capability for high performance pseudocapacitor applications.

In this paper, CoMn_2_O_4_@Co_3_O_4_ core/shell hybrid nanostructure arrays on Ni foam with unique hierarchical nanostructure for supercapacitor applications were successfully prepared *via* a facile hydrothermal method. The CoMn_2_O_4_@Co_3_O_4_ core/shell hybrid nanostructure arrays provides a large surface area and a number of electrochemical reactive sites, and faster ion diffusion and electron transport at electrode/electrolytes interface compared with single CoMn_2_O_4_ nanoneedles arrays or Co_3_O_4_ nanosheets electrode. Therefore, such interconnected core/shell hybrid network configuration can effectively increase capacitance and cycling stability. The CoMn_2_O_4_@Co_3_O_4_ hybrid electrodes presented herein exhibited remarkable electrochemical performance for SCs. The CoMn_2_O_4_@Co_3_O_4_//AC ASC device also exhibits high specific energy and energy density. These results suggest that the CoMn_2_O_4_@Co_3_O_4_ core/shell hybrid nanostructure arrays can act as a high performance electrode material for SCs applications.

## 2. Experimental

### 2.1. Materials

The urea, ethylene glycol, Co(NO_3_)_2_·6H_2_O, Mn(CH_3_COO)_2_·4H_2_O, NH_4_F and Co(CH_3_COO)_2_·4H_2_O are analytical grade without further purification and purchased from Zhanyun Chemical Co., Ltd., Shanghai, China. Besides, active carbon (AC) was purchased from Tanfeng Tech. Inc (Suzhou, China) with a surface area of 2100 m^2^ g^−1^.

### 2.2. Synthesis of CoMn_2_O_4_ nanoneedles arrays

In a typical process, firstly, commercially available Ni foam was pretreated with acetone, 3 M HCl solution, deionized water, and ethanol in sequence, and kept in vacuum oven at 60 °C for 6 h. To obtain a homogeneous precursor solution, 2.10 g of Co(NO_3_)_2_·6H_2_O, 3.48 g of Mn(CH_3_COO)_2_·4H_2_O, 5.07 g of urea and 1.63 g of NH_4_F were dissolved into the 70 mL ethanol under magnetic stirring. The total volume was then made up to 350 mL by adding distilled water and transferred to a Teflon-lined stainless steel autoclave. Then, the well-cleaned Ni foam (1 × 1 cm^2^ in sizes) was immersed in the autoclave. Subsequently, the autoclave was sealed and placed in an electrical oven at 160 °C for 16 h. After reaction and cooled to room temperature, the precursor deposited Ni foam was taken out and cleaned with ethanol and deionized water, then dried in a vacuum furnace at 60 °C for 6 h. The dried sample was then calcined in air at 400 °C with the heating rate of 10 °C min^−1^ and kept for 3 h (deposition weight = 3.5 mg cm^−2^).

### 2.3. Synthesis of CoMn_2_O_4_@Co_3_O_4_ core/shell hybrid nanostructure arrays

The CoMn_2_O_4_ nanoneedles arrays were used as the skeleton for the growth of Co_3_O_4_ nanosheets shell, which were synthesized by a simple hydrothermal method. Firstly, 0.8 g of Co(CH_3_COO)_2_·4H_2_O was were dissolved in 35 mL ethylene glycol/H_2_O (6 : 1) mixed solvent and stirring for 30 min at room-temperature. Then, 0.6 g of sodium dodecyl sulfate as a surfactant was added under stirring. Afterwards, this solution was transferred into a Teflon-lined stainless steel autoclave and heated in an oven at 180 °C for 12 h and then cooled to room temperature. Finally, the solid product was washed with deionized water. Then, the precursor was dried at 60 °C and calcined at 300 °C for 3 h in air to obtain CoMn_2_O_4_@Co_3_O_4_. The mass loading of CoMn_2_O_4_@Co_3_O_4_ was 4.6 mg, corresponding with the gain weight of Ni foam. For comparison, the sole Co_3_O_4_ nanosheets electrode growing on Ni foam directly was prepared with the same method described above.

### 2.4. Characterization

The product scratched down from the Ni foam were characterized by an X-ray diffractometer (XRD, X'pert MPD Pro, Philips, Netherlands) using Cu Ka radiation (*λ* = 1.5406 Å). The surface morphologies and micro-structures of the electrodes were characterized by field emission scanning electron microscopy (FESEM, Zeiss Supra 35VP, USA) and high resolution transmission electron microscopy (HRTEM, JEM- 2100, Japan). The specific surface area (BET method) and pore size distribution measurements were performed in a micromeritics ASAP 2020 sorptometer at 77 K.

### 2.5. Electrochemical measurements

The electrochemical performances were carried out on an electrochemical workstation (CHI660E, Shanghai Chen Hua Co. Ltd, China) using a three-electrode system, in which as-prepared sample was used as the working electrode, Pt wire as the counter electrode, a mercury oxide mercury electrode (Hg/HgO) as the reference electrode and freshly prepared 3 M KOH aqueous solution was used as the electrolyte, respectively. The cycling tests were conducted using a LAND battery program-control test system (CT2001A, Wuhan LANHE Co. Ltd, China).

### 2.6. Assembling of CoMn_2_O_4_@Co_3_O_4_//AC ASC

The prepared CoMn_2_O_4_@Co_3_O_4_ core/shell nanoflowers electrode (positive) and the charge balanced activated carbon (AC) electrode (negative) were pressed together and separated by a fibrin separator (140 μm thick). The electrodes and separator were immersed in an aqueous 3 M KOH electrolyte and assembled layer by layer with soft polyethylene terephthalate (PET) membranes at room temperature.

The specific capacitance was determined from galvanostatic charge/discharge *via*eqn (1):

1 *C*_m_= *I*Δ*t*/*m*Δ*V*

where *C*_m_ is the specific capacitance of the active material (F g^−1^), *I* (A), Δ*t* (s), Δ*V* (V) and *m* (g) represent the applied current, total discharge time, potential window and the mass of the active materials, respectively. The energy and power density of the ASC devices were calculated as follows:

2 *E* = *C*_m_Δ*V*^2^/2

3 *P* = *E*/Δ*t*

where *E* (W h kg^−1^) is the average energy density; *C*_m_ (F g^−1^) is the specific capacitance of the ASC device; Δ*V* (V) is the voltage window; *P* (W kg^−1^) is the average power density and Δ*t* (s) is the discharge time.

## 3. Results and discussions

### 3.1. Characterization of CoMn_2_O_4_@Co_3_O_4_ core/shell electrodes

**Fig. 1:**
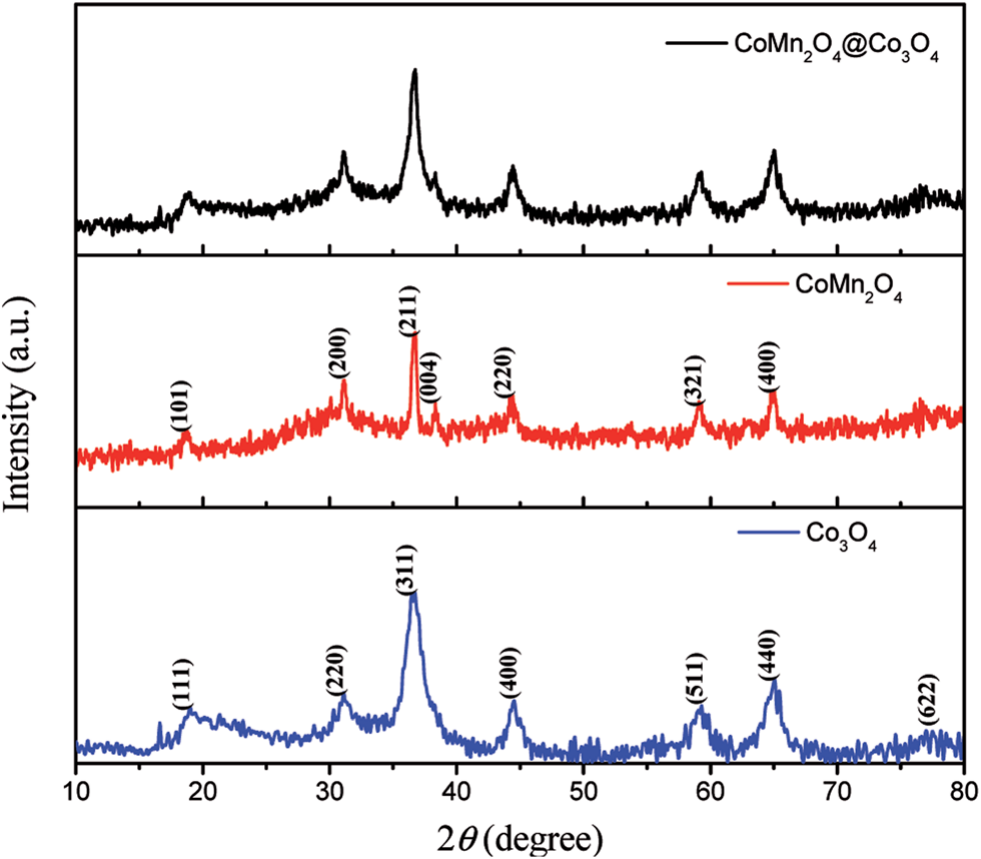
XRD patterns of CoMn_2_O_4_@Co_3_O_4_, CoMn_2_O_4_ and Co_3_O_4_ core/shell arrays.

The XRD patterns of as-synthesized CoMn_2_O_4_, Co_3_O_4_ and CoMn_2_O_4_@Co_3_O_4_ exfoliated from Ni foam are shown in Fig. 1. The patterns of as-synthesized samples could be indexed to the CoMn_2_O_4_ (JCPDS Card no. 77-0471)^24^ and Co_3_O_4_ (JCPDS Card no.42-1467).^25^ Besides, from the pattern of CoMn_2_O_4_@Co_3_O_4_, CoMn_2_O_4_ and Co_3_O_4_ diffraction peaks overlap together and broad peaks were recognized, indicating the combination of the crystalline CoMn_2_O_4_ and Co_3_O_4_ in one nanostructure. Energy Dispersive Spectrometer (EDS) analysis datum, SEM-mapping pictures and STEM mapping images shown in Fig. S1† is consistent with the XRD result. It shows that as-synthesized CoMn_2_O_4_@Co_3_O_4_ is mainly composed of Co, O, and Mn chemical elements.

**Fig. 2:**
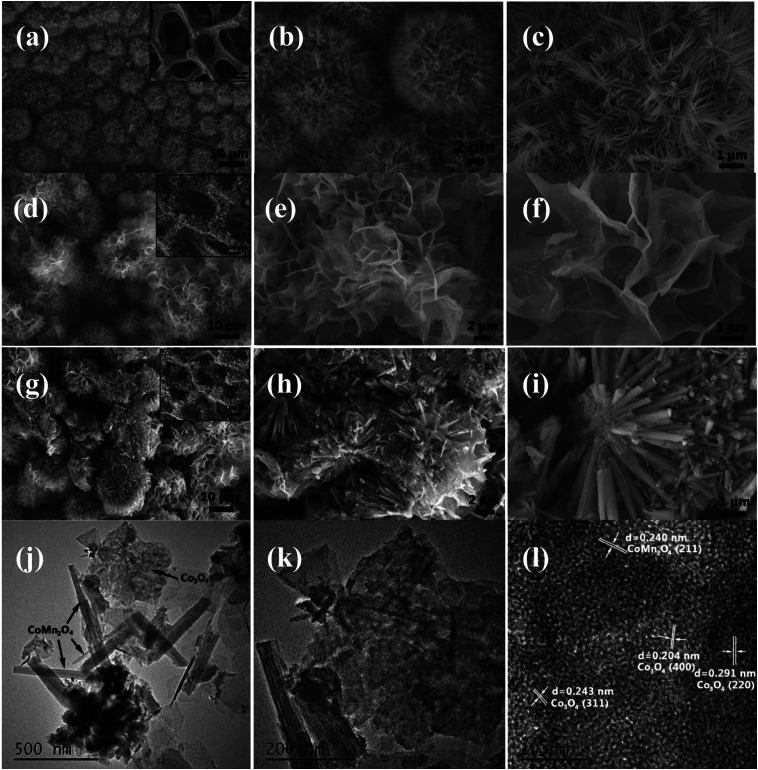
(a–c) SEM images of CoMn_2_O_4_ nanoneedles arrays. (d–f) SEM images of Co_3_O_4_ nanoflowers. (g–i) CoMn_2_O_4_@Co_3_O_4_ core/shell nanoflower arrays. (j–l) TEM image of CoMn_2_O_4_@Co_3_O_4_.

The structure and morphology of the as-prepared samples were characterized by FESEM and TEM. Fig. 2(a–c) present the typical FESEM images of the CoMn_2_O_4_ nanoneedles arrays supported on Ni foam. On the observation of high-magnification image (Fig. 2(c)), we found that the obtained CoMn_2_O_4_ nanoneedles arrays (with lengths of ∼1μm and diameters of ∼20–70 nm) are highly densely. Fig. 2(d–f) show FESEM images of the as-synthesized large-scale and dense Co_3_O_4_ nanosheets. It can be observed that the Co_3_O_4_ nanosheets exhibits interconnected big channels. Fig. 2(g–i) represent the FESEM images of the CoMn_2_O_4_@Co_3_O_4_ core–shell hybrid nanostructure arrays grown on Ni foam. The CoMn_2_O_4_ nanoneedles are uniformly enwrapped in the Co_3_O_4_ nanosheets. The CoMn_2_O_4_ nanoneedles clusters were almost all covered by Co_3_O_4_ nanosheets with an overall size of ∼15 μm. The TEM images in Fig. 2(j–l) also further reveal the hybrid nanostructure of an individual CoMn_2_O_4_@Co_3_O_4_ core/shell nanowire, although hierarchical nanoflower structure cannot be clearly observed, probably owing to the partial damage from TEM sample preparation procedure (sonication). The HRTEM image of CoMn_2_O_4_@Co_3_O_4_ further verifies the crystal structure. Fig. 2(l) and S2† reveal a distinct set of visible lattice fringes with interplanar spacings of 0.240 nm, 0.453 nm, 0.291 nm, 0.243 nm and 0.204 nm, which correspond to the (211) plane of CoMn_2_O_4_, (111) plane of Co_3_O_4_, (220) plane of Co_3_O_4_, (311) plane of Co_3_O_4_, and (400) plane of Co_3_O_4_, respectively. The results are consistent with the XRD results patterns. The N_2_-adsorption/desorption measurements were carried out to study the surface area and porosity property of the samples. BET analysis results show that the specific surface area of CoMn_2_O_4_@Co_3_O_4_ core/shell nanoflowers is 67.5 m^2^ g^−1^, which is much higher than that of CoMn_2_O_4_ nanoneedles (44.3 m^2^ g^−1^) and Co_3_O_4_ nanoflowers (61.2 m^2^ g^−1^) (Fig. S3†). The pore size distributions of these samples are shown in the insets, confirming that the samples have mesoporous characteristics. Obviously, the CoMn_2_O_4_@Co_3_O_4_ core/shell configuration can provide a higher surface area, which is mainly attributed to the interconnected Co_3_O_4_ nanosheets and the aligned CoMn_2_O_4_ nanoneedles scaffold that creating a 3D interconnected porous network and highly porous surface morphology. Such interconnected network configuration not only provides large surface area for charge storage but also facilitates electrolyte penetration through the mesopores and increase the utilization of the active materials.^26–29^ Based on the above mentioned merits, the CoMn_2_O_4_@Co_3_O_4_ core/shell nanoflowers can be employed as excellent electrode material for SCs and the detailed electrochemical measurements are performed as follows.

### 3.2. Electrochemical performance of CoMn_2_O_4_@Co_3_O_4_ core/shell electrodes

**Fig. 3:**
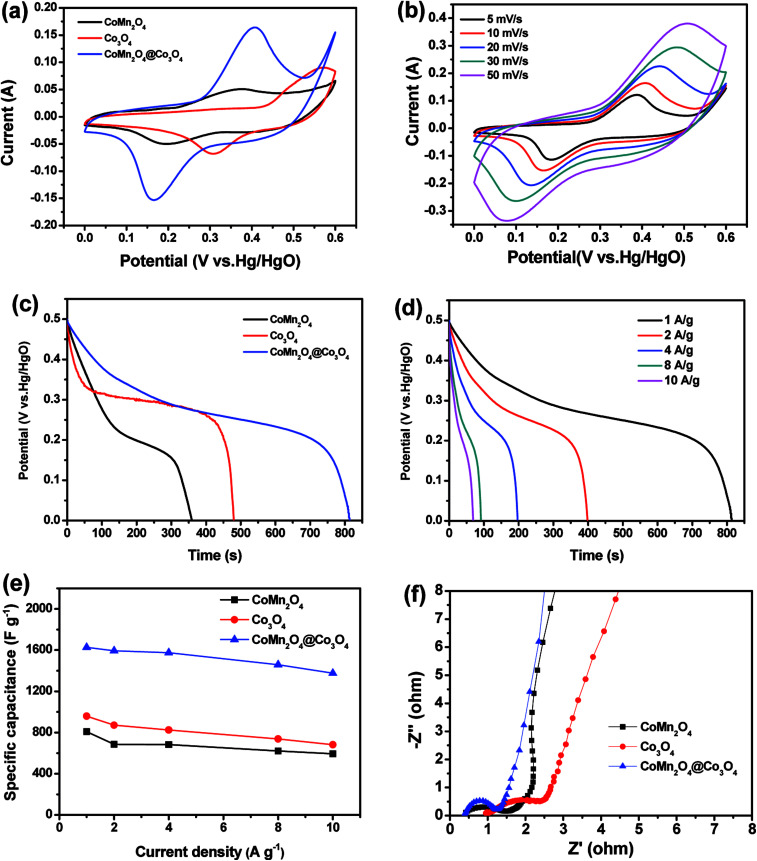
(a) CV curves of CoMn_2_O_4_, Co_3_O_4_ and CoMn_2_O_4_@Co_3_O_4_ electrodes at a scan rate of 10 mV s^−1^. (b) CV curves of the CoMn_2_O_4_@Co_3_O_4_ electrode at various scan rates. (c) Discharge curves of three electrodes at a current density of 1 A g^−1^. (d) Discharge curves of the CoMn_2_O_4_@Co_3_O_4_ core/shell electrode at different current densities. (e) Specific capacitance of three electrodes at various current densities. (f) Nyquist plots of CoMn_2_O_4_, Co_3_O_4_ and CoMn_2_O_4_@Co_3_O_4_ electrodes measured in the frequency range from 100 kHz to 0.01 Hz.

Fig. 3(a) shows the CV curves of CoMn_2_O_4_, Co_3_O_4_ and CoMn_2_O_4_@Co_3_O_4_ core/shell electrodes between a potential window of 0 and 0.6 V at a scan rate of 10 mV s^−1^. Clearly, the CV integrated area of the CoMn_2_O_4_@Co_3_O_4_ electrode was significantly larger compared with the unitary electrodes, indicating that the CoMn_2_O_4_@Co_3_O_4_ core/shell electrodes have a apparently larger specific capacitance than pure CoMn_2_O_4_ or Co_3_O_4_ electrode.

The redox reaction mechanism for CoMn_2_O_4_ is represented by the following equations:^30–33^

4 CoMn_2_O_4_ + H_2_O + OH^−^ → CoOOH + 2MnOOH + e^−^

5 MnOOH + OH^−^ → MnO_2_ + H_2_O + e^−^

As shown, two pairs of redox peaks for the Co_3_O_4_ electrode appear in the CVs, which is due to the Co^2+^/Co^3+^ and Co^3+^/Co^4+^ reactions, described by the following reaction:^30,34^

6 (+)3Co(OH)_2_ + OH^−^ → Co_3_O_4_ + 4H_2_O + e^−^

7 Co_3_O_4_ + OH^−^ + H_2_O → 3CoOOH + e^−^

8 CoOOH + OH^−^ → CoO_2_ + H_2_O + e^−^

9 4OH^−^ → O_2_ + 2H_2_O + 4e^−^

10 (−)CoO_2_ + H_2_O + e^−^ → CoOOH + OH^−^

11 3CoOOH + e^−^ → Co_3_O_4_ + OH^−^ + H_2_O

12 Co_3_O_4_ + H_2_O + 2e^−^ → 3Co(OH)_2_ + 2OH^−^

A pair of significantly enhanced redox peaks can be observed at the voltage of 0.16 V and 0.41 V for the CoMn_2_O_4_@Co_3_O_4_ electrode, which may originate from the fast faradaic redox reactions and the short ion diffusion path provided by CoMn_2_O_4_ and Co_3_O_4_, as shown in reaction (4)–(12).

Fig. 3(b) shows the CV curves of the CoMn_2_O_4_@Co_3_O_4_ electrode at various scan rates. As the sweep rate increases, the cathodic peak position was shifted from 0.18 to 0.07 V and the anode peak position was shifted from 0.38 to 0.51 V, which is due to the polarization effect of the electrode.

The improved capacitive performance was also evaluated by galvanostatic charge–discharge (GCD) measurements over a potential range from 0 to 0.5 V. Fig. 3(c) presents the comparison of the discharge curves at 1 A g^−1^ for CoMn_2_O_4_, Co_3_O_4_, and CoMn_2_O_4_@Co_3_O_4_ electrodes. As expected, the CoMn_2_O_4_@Co_3_O_4_ electrode demonstrate a much longer discharging time than the CoMn_2_O_4_ and Co_3_O_4_ electrodes, indicating its higher capacitance. In addition, the discharge curves of the CoMn_2_O_4_@Co_3_O_4_ electrode and specific capacitance of the samples from 1 to 10 A g^−1^ are shown in Fig. 3(d) and (e), respectively. Based on the GCD curves, the capacitances of CoMn_2_O_4_@Co_3_O_4_ electrode are calculated to be 1627, 1593, 1575, 1459 and 1376 F g^−1^ at 1, 2, 4, 8 and 10 A g^−1^, respectively, which are much higher than the pure CoMn_2_O_4_ electrode with the value of 808, 686, 684, 621 and 593 F g^−1^ and bare Co_3_O_4_ electrode with the value of 959, 871, 824, 738 and 682 F g^−1^ at the same discharge current densities. The significant performance increment of the CoMn_2_O_4_@Co_3_O_4_ hybrid electrode was also confirmed by electrochemical impedance spectroscopy (EIS). The impedance spectra were obtained using an AC voltage of 5 mV in a frequency range from 0.01 Hz to 100 kHz. The electrochemical impedance data were analyzed with a Randles equivalent circuit that includes the charge transfer resistance (*R*_ct_), and the straight line in the low-frequency region represents the diffusive resistance (*W*). In addition, the internal resistance (*R*_e_) is obtained from the high-frequency intersection of the Nyquist plot in the real axis.^35^ There are a single semicircle in the high-frequency region and a straight line in the low-frequency region in the Nyquist plots of the three electrodes (Fig. 3(f)). The semicircle at high frequency region corresponds to the charge transfer processes whereas the straight line at low-frequency region relates to the ion diffusion processes.^36^ The smaller semicircle diameter (lower *R*_ct_) indicates faster electron-transfer kinetics of corresponding electrode.^37–39^ CoMn_2_O_4_@Co_3_O_4_ electrode exhibits a smaller radius in the high-frequency region and a steeper line in the low-frequency region than those of other two electrodes, indicating superior charge transfer and ion diffusion kinetics behavior.

**Fig. 4:**
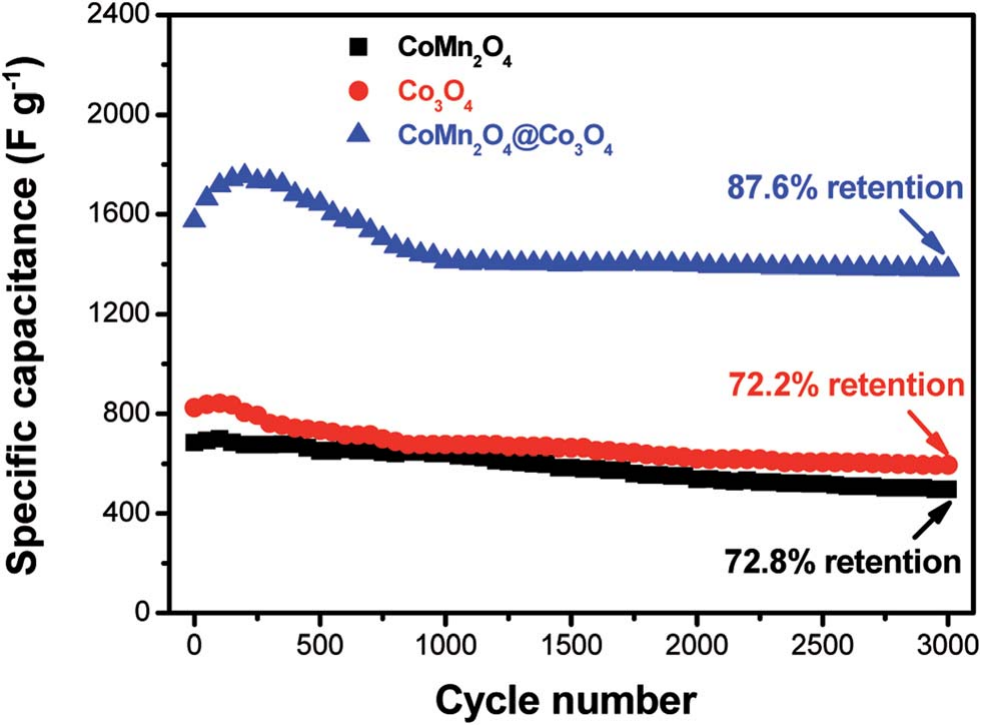
Cycling performances of CoMn_2_O_4_, Co_3_O_4_ and CoMn_2_O_4_@Co_3_O_4_ electrodes at a current density of 4 A g^−1^.

Cycling stability is another important parameter for high-performance supercapacitors. The long-term cycling stability of as-synthesized supercapacitors was tested through a repetitive galvanostatic charge/discharge process at a constant current density of 4 A g^−1^ for 3000 cycles (Fig. 4). It can be observed that the specific capacitance of CoMn_2_O_4_@Co_3_O_4_ electrode firstly increases before 200 cycles, and then decreases with cycle number increasing, suggesting an activation process occurring during the beginning of a successive scan.^40^ It is important to indicate that the electrode shows capacitance retention of 87.6% after 3000 cycles. The decrease of the specific capacitance could be attributed to the dissolution of the outer Co_3_O_4_ in alkaline electrolyte, leading to loss of active materials. The excellent stability may be mainly attributed to the unique hierarchical porous core/shell morphology of the CoMn_2_O_4_@Co_3_O_4_ hybrid. The CoMn_2_O_4_ nanowire arrays on the Ni foam serve as a supporting framework for the ultrathin and interconnected Co_3_O_4_ nanosheets to produce an hierarchical nanostructures array and therefore enhance the mechanical stability.

In conclusion, the outstanding electrochemical performance of the CoMn_2_O_4_@Co_3_O_4_ hybrid electrode can be mainly ascribed to the unique core–shell hierarchical porous nanostructure, providing the advantages as follows: (i) both CoMn_2_O_4_ and Co_3_O_4_ are good pseudo-capacitive materials in KOH electrolyte, hence contributing to the enhanced pseudo-capacitance activity. (ii) The unique hierarchical core/shell porous nanostructure is anticipated to enhance the amount of accessible active sites for the capacitive reactions,^41^ and can supply a short and fast ion diffusion pathway, thus enhancing the faradic reaction.

### 3.3. Electrochemical characterization of CoMn_2_O_4_@Co_3_O_4_//AC ASC

**Fig. 5:**
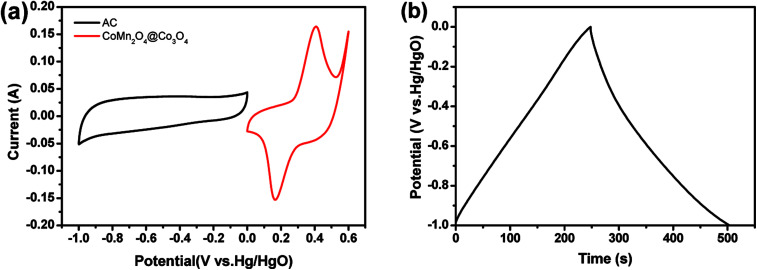
(a) CV curves of the CoMn_2_O_4_@Co_3_O_4_ and AC electrodes at a scan rate of 10 mV s^−1^. (b) Galvanostatic charge–discharge curve of the AC electrode at a current density of 1 A g^−1^.

**Fig. 6:**
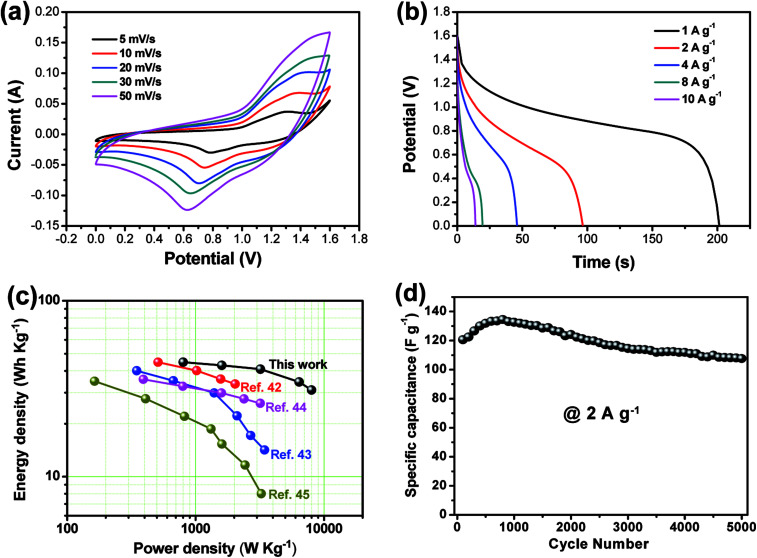
(a) CV curves of the CoMn_2_O_4_@Co_3_O_4_//AC electrode at various scan rates. (b) Discharge curve of the CoMn_2_O_4_@Co_3_O_4_//AC electrode at a current density of 1 to 10 A g^−1^. (c) Ragone plot of the CoMn_2_O_4_@Co_3_O_4_//AC device. The values reported for others devices based on core–shell nanostructure materials are given here for a comparison. (d) Cycling performances of the CoMn_2_O_4_@Co_3_O_4_//AC electrode at a current density of 2 A g^−1^.

As shown in Fig. 5(a), the CV curve of AC electrode from −1.0 to 0 V (*vs.* Hg/HgO) exhibits a nearly rectangular shape, typical of capacitive behavior, which indicates the characteristic of the electric double layer capacitance, while that of CoMn_2_O_4_@Co_3_O_4_ within a voltage the potential window of 0 to 0.6 V (*vs.* Hg/HgO). As shown in Fig. 6(a), the CoMn_2_O_4_@Co_3_O_4_//AC device showed a nearly ideal capacitive behavior with a cell voltage up to 1.6 V at a scan rate of 50 mV s^−1^. The achieved high operating voltage of the device benefits from the advantage of the different stable potential windows of CoMn_2_O_4_@Co_3_O_4_ and AC electrodes.

To obtain the maximum performance of the CoMn_2_O_4_@Co_3_O_4_//AC ASC, it is crucial to keep the charges balanceable with the relationship *q*+ = *q*^−^. In order to get the charge balanceable, the optimum loading mass of AC was decided by the following equation:

13 *m*_(CoMn_2_O_4_@Co_3_O_4_)_/*m*_(AC)_ = *C*_m(AC)_Δ*V*_(AC)_/*C*_m(CoMn_2_O_4_@Co_3_O_4_)_Δ*V*_(CoMn_2_O_4_@Co_3_O_4_)_

where *m* is the mass of activated material, *C*_m_ represents the specific capacitance and Δ*V* is the potential window in the three-electrode test system. The charge/discharge curve of the AC electrode at 1 A g^−1^ is depicted in Fig. 5(b) and the calculated *C*_m_ value of the AC electrode is 254 F g^−1^. The *C*_m_ value of the CoMn_2_O_4_@Co_3_O_4_ electrode is 1627 F g^−1^ at 1 A g^−1^ (Fig. 3(e)). The mass loading of AC in CoMn_2_O_4_@Co_3_O_4_//AC was 17.7 mg.

Fig. 6(a) shows the CV curves of the CoMn_2_O_4_@Co_3_O_4_//AC ASC at various scan rates. The CoMn_2_O_4_@Co_3_O_4_//AC ASC exhibits an irregular shape CV curve, which is distinct from the CV curve of the AC electrode with a rectangular shape. The distortion of CV curves from rectangular-like shapes may be attributed to the pseudo-capacitance from CoMn_2_O_4_@Co_3_O_4_//AC cathode.^42,43^ The discharge curves of the ASC device at various current densities within the potential window of 0–1.6 V are shown in Fig. 6(b). The specific capacitances of CoMn_2_O_4_@Co_3_O_4_//AC ASC were calculated according to the eqn (1) to be 125.8, 120.6, 114.7, 97.1 and 87.4 F g^−1^ at the current densities of 1, 2,4, 8, 10 A g^−1^, respectively. It is worth noting that even at a high current density of 10 A g^−1^, the specific capacitance still reaches up to 87.4 F g^−1^ (about 69.5% of the capacitance retained), indicating its good rate capability. Note that the specific capacitance is about six times larger than that of conventional AC-based symmetric capacitors (∼20 F g^−1^),^44^ which is enhanced by the ultra-high pseudo-capacitance of the CoMn_2_O_4_@Co_3_O_4_ electrode. To examine the supercapacitor performance of the CoMn_2_O_4_@Co_3_O_4_//AC device, the energy density (*E*) and power density (*P*) of the ASC were calculated according to the eqn (2) and (3), and corresponding Ragone plot is in Fig. 6(c). The CoMn_2_O_4_@Co_3_O_4_//AC supercapacitor could achieve a high *E* value of 44.8 W h kg^−1^ (with a *P* value of 800.5 W kg^−1^) and still maintain 31.1 W h kg^−1^ at a high power density of 8010.3 W kg^−1^. Note that the energy and power densities of the present ASC are superior to that of many ASCs using core–shell nanostructure materials as electrode positive electrode, such as MnMoO_4_·H_2_O@MnO_2_//AC,^45^ Co_3_O_4_@Ni(OH)_2_//AC,^46^ NiCo_2_S_4_@Co(OH)_2_//AC^47^ and NiCo_2_O_4_@MnO_2_//AC.^48^ We believe that the high energy and power density of our ASC are mainly due to the wide working voltage window and the improved specific capacitance comes from the high synergistic effect of these two electrodes.

The long-term cycling stability of the CoMn_2_O_4_@Co_3_O_4_//AC ASC was examined by successive galvanostatic charge–discharge cycling at a current density of 2 A g^−1^ for 5000 cycles. As shown in Fig. 6(d), the ASC shows capacitance retention of 89.2% after 5000 cycles. Note that the rate capability and cycling stability of the CoMn_2_O_4_@Co_3_O_4_//AC ASC is comparable or superior to many state-of-art ASC systems (Table S2†), further indicating the advantage of the CoMn_2_O_4_@Co_3_O_4_//AC ASC. The high power density with energy density, excellent rate capability and cycling stability make the CoMn_2_O_4_@Co_3_O_4_//AC ASC as a promising candidate for practical energy storage.

## 4. Conclusions

We have successfully prepared hierarchical CoMn_2_O_4_@Co_3_O_4_ core/shell nanowire arrays with attractive pseudocapacitance behaviors *in situ* grown on Ni foam by simple, cost-effective, and facile hydrothermal method. The resultant CoMn_2_O_4_@Co_3_O_4_ core/shell nanowire arrays exhibited significant capacitance, as compared with bare CoMn_2_O_4_ and Co_3_O_4_. The CoMn_2_O_4_@Co_3_O_4_ hybrid with unique architecture is then used as binder-free electrode for supercapacitors. This electrode exhibits a high specific capacitance of 1627 F g^−1^ at 1 A g^−1^ and 1376 F g^−1^ at 10 A g^−1^. An ASC device employing the CoMn_2_O_4_@Co_3_O_4_ electrode and active carbon electrode delivers a high specific energy of 125.8 F g^−1^ at 1 A g^−1^ as well as high energy density of 44.8 W h kg^−1^. Also, the ASC exhibits high cycling stability with capacitance retention of 89.2% after 5000 charge/discharge cycles at a current density of 2 A g^−1^. The superior electrochemical performance indicate that the present low-cost CoMn_2_O_4_@Co_3_O_4_ core/shell hybrid can serve as a promising electrode material for high-performance supercapacitors.

## Conflicts of interest

The authors declare no conflict of interest.

## Supplementary Material

RA-008-C8RA06289G-s001
