# Supplementary material for: Advanced binder-free electrodes based on CoMn2O4@Co3O4 core/shell nanostructures for high-performance supercapacitors
Source: RSC Adv. 2018 Sep 10;8(55):31594–602. doi: 10.1039/c8ra06289g (PMC9085651; doi:10.1039/c8ra06289g)
Supplement: RA-008-C8RA06289G-s001 [file RA-008-C8RA06289G-s001.pdf]

Electronic Supplementary Material (ESI) for RSC Advances.
This journal is © The Royal Society of Chemistry 2018

Electronic Supplementary Information

Advanced binder-free electrode based on $CoMn_2O_4@Co_3O_4$ core/shell nanostructures for high-performance supercapacitors

Xiaobo Chen [1,*] Xiao Liu[1], Yongxu Liu[1], Yameng Zhu[1], Guoce Zhuang [1], Wei Zheng [1], Zhenyu Cai [1], Peizhi Yang [2,*]

1 School of New Energy and Electronic Engineering, Yancheng Teachers University, Yancheng, 224051, PR China

2 Key Laboratory of Education Ministry for Advance Technique and Preparation of Renewable Energy Materials, Yunnan Normal University, Kunming, 650500, PR China

E-mail addresses: chenxbok@126.com (X. Chen); pzhyang@hotmail.com (P. Yang)

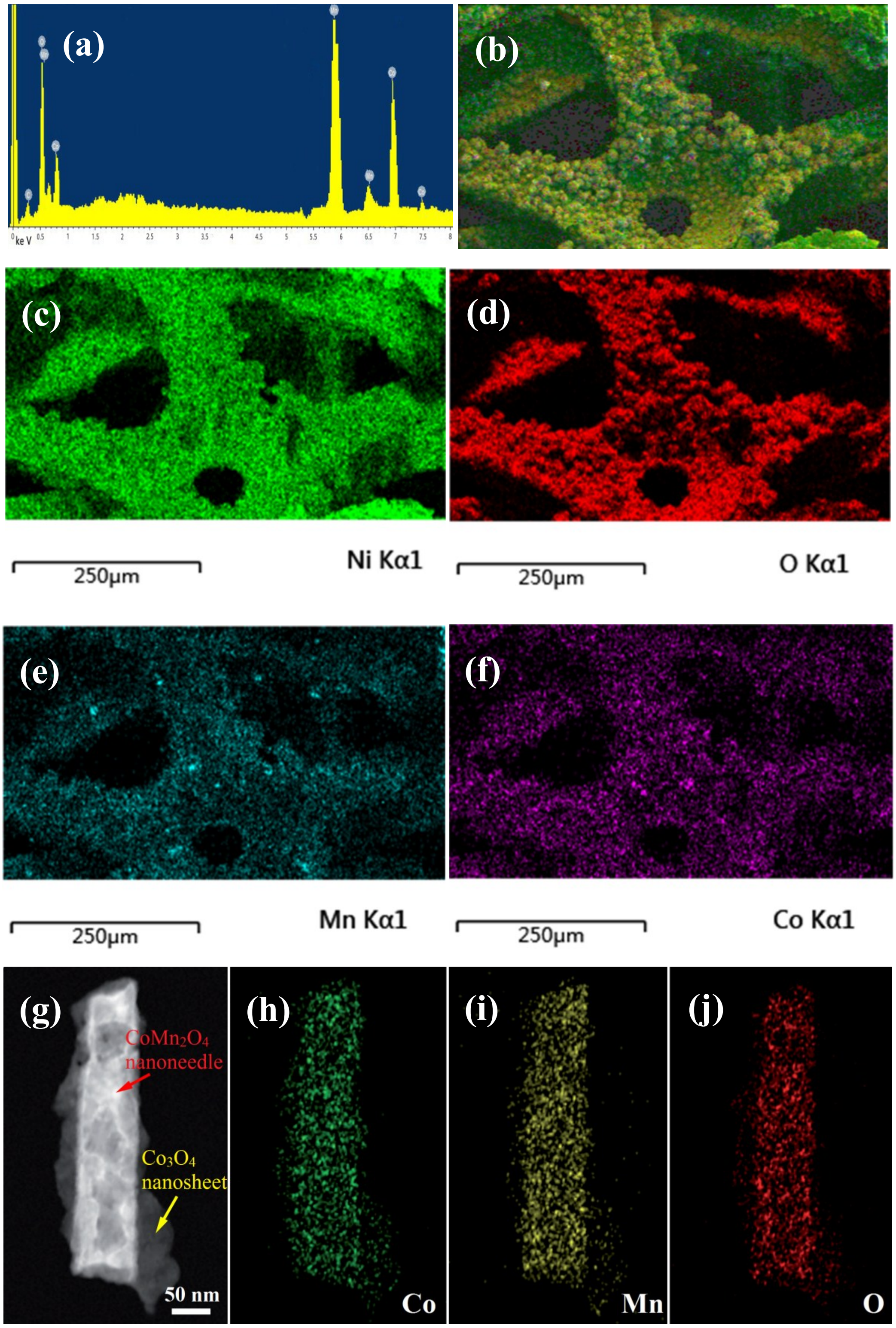

Fig. S1 EDS spectrum (a) and elemental mapping (b-f) of $CoMn_2O_4@Co_3O_4$ core–shell arrays. (g) STEM image and (h-j) corresponding elemental mapping images of $CoMn_2O_4@Co_3O_4$.

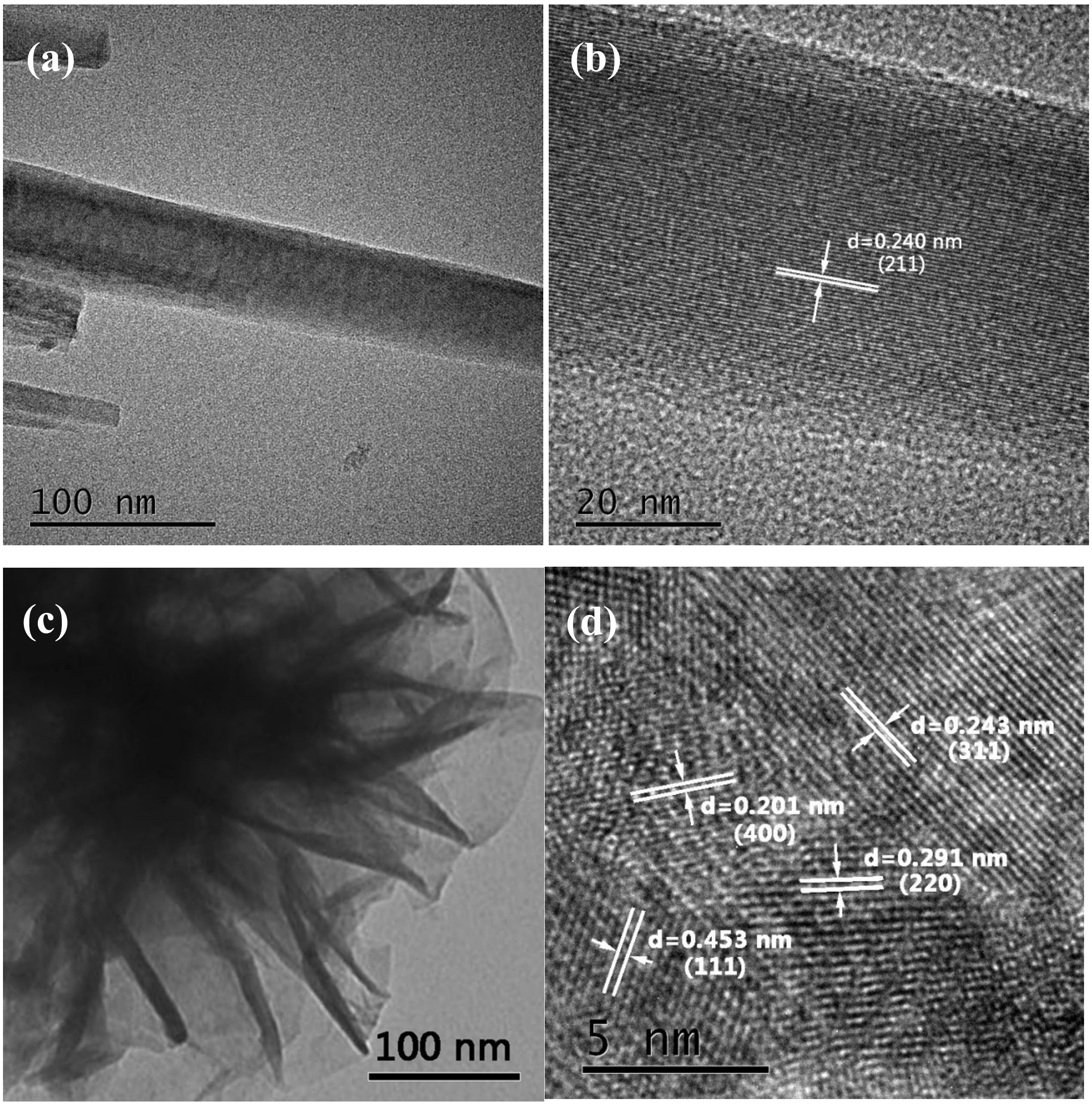

Fig. S2 (a,b) TEM images of $CoMn_2O_4$ nanoneedle. (c,d) TEM images of Co_3O_4 nanoflower.

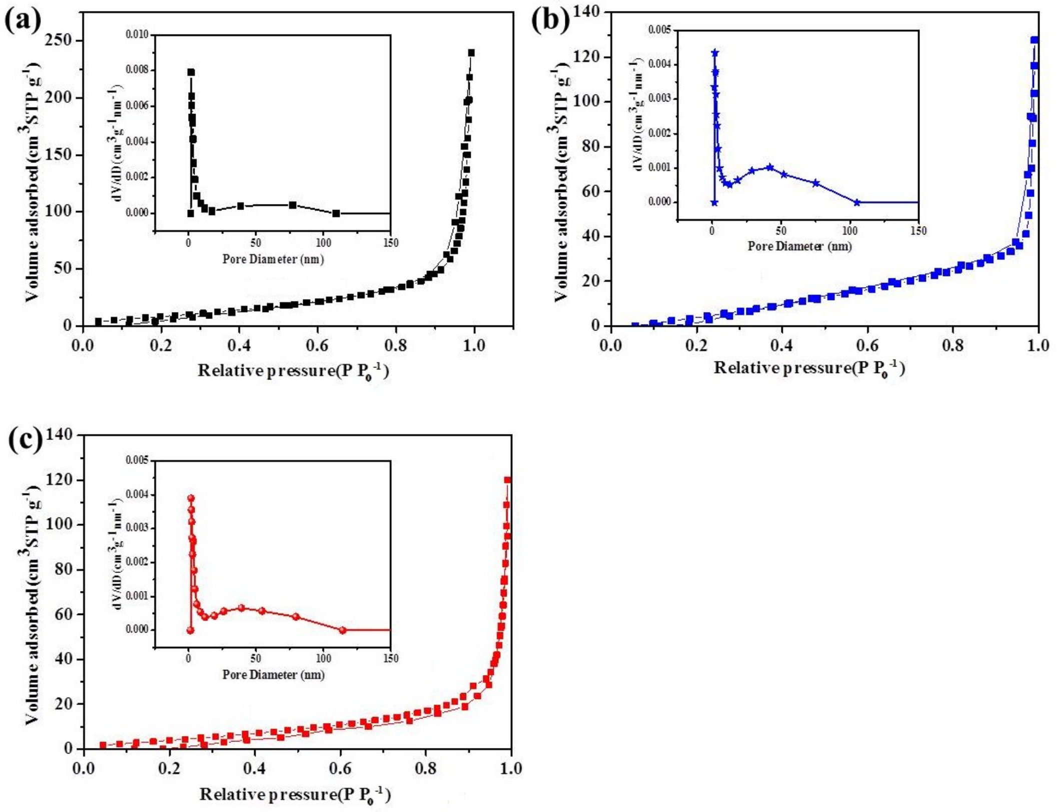

Fig. S3 N_2 adsorption and desorption isotherms of (a) $CoMn_2O_4$ nanoneedles; (b) Co_3O_4 nanoflowers; (c) $CoMn_2O_4$@Co_3O_4 core/shell nanoflower. The insets show corresponding pore size distributions.

Table S1. Comparison of specific capacitances and cycling performance of the reported $CoMo_2O_4$ or Co_3O_4 oxides based electrodes and the present work.

**Electrode materials**	**Current density (A g^{-1} or mA cm^{-2})**	**Capacitance F g^{-1}**	**Cycles**	**Retention**	**Ref.**
Hierarchically porous Co_3O_4 film	2 A g^{-1}	352	2500	82.6%	[1]
$CoMoO_4$•0.9H_2O nanorods	10 mA cm^{-2}	293	1000	96 %	[2]
Cobalt monoxide nanowire @ nickel hydroxidenitrate nanoflake	4.55 A g^{-1}	307	2000	95.1 %	[3]
Porous Co_3O_4 nanowires	5 A g^{-1}	250	2000	98 %	[4]
$CoMoO_4$-$NiMoO_4$•xH_2O bundles	50mA cm^{-2}	826	1000	75.1%	[5]
$CoMoO_4$/graphene composites	1 A g^{-1}	394.5	500	78.4%	[6]
$CoMoO_4$ nanorods	5 mA cm^{-2}	202	2000	97.5 %	[7]
$CoMoO_4$ nanoplate arrays	12 mA cm^{-2}	787	4000	73.6%	[8]
Polyaniline-wrapped 1D $CoMoO_4$•0.75H_2O nanorods	1 A g^{-1}	380	1000	90.4%	[9]
$CoMn_2O_4$ nanosheet	5 A g^{-1}	1267	3000	100%	[10]
Co_3O_4@MnO_2 nanowire arrays	0.2 A g^{-1}	560	5000	95 %	[11]
Co_3O_4@NiO hierarchical nanowire arrays	1 A g^{-1}	1236.67	5000	88.37%	[12]
CWs-Co3O4 composite	0.5 A g^{-1}	978.9	2000	94.5%	[13]
PANI-Co3O4 nanocomposites	1.25 A g^{-1}	1184	1000	84.9%	[14]
**$CoMn_2O_4$@Co_3O_4**	**4A g^{-1}**	**1575**	**3000**	**87.6 %**	**This work**

Table S2. A comparison of rate capability and cycling stability for different asymmetric supercapacitors

**Supercapacitors**	**Rate capability (retention %)**	**Cycling stability (retention %)**	**Ref.**
$NiCo_2O_4$//AC	46% (0.5→8 A g^{-1})	64% (5,000 cycles, 1.5 A g^{-1})	[15]
MnO_2//AC	72% (0.25→4A g^{-1})	94% (5,000 cycles, 1 A g^{-1})	[16]
Ni-P@$NiCo_2O_4$//AC	64% (1→16 A g^{-1})	78% (10,000 cycles, 4 A g^{-1})	[17]
$FeCo_2O_4$@MnO_2//AC	/	90% (5,000 cycles, 25 mA cm^{-2})	[18]
$MnMoO_4\cdot H_2O$@MnO_2 //AC	75% (5→20 mV s^{-1})	85% (5,000 cycles, 50 mA g^{-1})	[19]
Co_3O_4//AC	/	90% (4,000 cycles, 5 A g^{-1})	[20]
CoO@Co3O4//graphene	55% (1→15 A g^{-1})	75% (6,000 cycles, 3 A g^{-1})	[21]
**$CoMn_2O_4$@Co_3O_4//AC**	**70% (1→10 A g^{-1})**	**89% (5,000 cycles, 2 A g^{-1})**	**This work**

References

1. Duana BR, Cao Q (2012) Electrochimica Acta 64: 154-161.
2. Liu MC, Kong LB, Ma XJ, Lu C, Li XM, Luo YC, Kang L (2012) New J Chem 36**:** 1713-1716.
3. Guan C, Liu JP, Cheng CW, Li HX, Li XL, Zhou WW, Zhang H, Fan HJ (2012) Energy Environ Sci 4: 4496-4499.
4. Wang B, Zhu T, Wu HB, Xu R, Chen JS, Lou (David) XW (2012) Nanoscale 4: 2145-2149.
5. Liu MC, Kong LB, Lu C, Ma XJ, Li XM, Luo YC, Kang L (2013) J Mater Chem A 1: 1380-1387.
6. Xia XF, Lei W, Hao QL, Wang WJ, Wang X (2013) Electrochim Acta 99: 253-261.
7. Liu MC, Kong LB, Lu C, Li XM, Luo YC, Kang L (2013) Mater Lett 94**:** 197-200.
8. Guo D, Zhang HM, Yu XZ, Zhang M, Zhang P, Li QH, Wang TH (2013) J Mater Chem A 1: 7247-7254.
9. Mandal M, Ghosh D, Giri S, Shakirb I, Das CK (2014) RSC Adv 4: 30832-30839.
10. Fu Y, Lu X, Zhao W, Zhang Y, Yang Y, Quan H, Xu X, Wang F (2015) Appl Surf Sci 357: 2013-2021.
11. Huang M, Zhang Y, Li F, Zhang L, Wen Z, Liu Q (2014) J Power Sources 252: 98-106.
12. Hu Q, Gu Z, Zheng X, Zhang X (2016) Chem Eng J 304: 223-231.
13. Zheng Y, Li Z, Xu J, Wang T, Liu X, Duan X, Ma Y, Zhou Y, Pei C (2016) Nano Energy 20: 94-107.
14. Hai Z, Gao L, Zhang Q, Xu H, Cui D, Zhang Z, Tsoukalas D, Tang J, Yan S, Xue C (2016) Appl Surf Sci 361: 57-62.
15. Ding R, Qi L, Jia M, Wang H (2013) Electrochim Acta 107: 494-502.
16. Li H, Zhang X, Ding R, Qi L, Wang H (2013) Electrochim Acta 108: 497-505.
17. Li X, Ding R, Yi L, Shi W, Xu Q, Liu E (2016) Electrochim Acta 222: 1169-1175.
18. Zhu F, Liu Y, Yan M, Shi W (2018) J Colloid Interf Sci 512: 419-427.
19. Xu J, Sun Y, Lu M, Wang L, Zhang J, Qian J, Liu X (2018) Chem Eng J 334: 1466-1476.
20. Guo D, Song X, Li F, Tan L, Ma H, Zhang L, Zhao Y (2018) Colloid Surface A 546: 1-8.
21. Cheng M, Duan S, Fan H, Su X, Cui Y, Wang R (2017) Chem Eng J 327: 100-108.
